# Dual mTOR inhibitor MLN0128 suppresses Merkel cell carcinoma (MCC) xenograft tumor growth

**DOI:** 10.18632/oncotarget.5878

**Published:** 2015-10-23

**Authors:** Aarthi Kannan, Zhenyu Lin, Qiang Shao, Stephanie Zhao, Bin Fang, Mauricio A. Moreno, Emre Vural, Brendan C. Stack, James Y. Suen, Krishnaswamy Kannan, Ling Gao

**Affiliations:** ^1^ Department of Dermatology, University of Arkansas for Medical Sciences, Little Rock, AR 72205, USA; ^2^ Department of Otolaryngology-Head and Neck Surgery, University of Arkansas for Medical Sciences, Little Rock, AR 72205, USA; ^3^ Department of Internal Medicine, University of Arkansas for Medical Sciences, Little Rock, AR 72205, USA; ^4^ Current address: Cancer Center, Union Hospital, Tongji Medical College, Huazhong University of Science and Technology, Wuhan 430000, China; ^5^ Current address: Critical Care Medicine, the First Affiliated Hospital of Nanchang University, Nanchang, Jiangxi 330006, China; ^6^ Pulaski Academy, Little Rock, AR 72212, USA; ^7^ Current address: Biomolecular Integrations, Little Rock, AR 72207, USA

**Keywords:** Merkel cell carcinoma, mTOR pathway, mTOR inhibitor, MLN0128

## Abstract

Merkel cell carcinoma (MCC) is an aggressive neuroendocrine skin cancer. Pathologic activation of PI3K/mTOR pathway and elevated expression of c-Myc are frequently detected in MCC. Yet, there is no targeted therapy presently available for this lethal disease. Recently, MLN0128, a second-generation dual TORC1/2 inhibitor is shown to have therapeutic efficacy in preclinical studies. MLN0128 is currently in clinical trials as a potential therapy for advanced cancers. Here we characterize the therapeutic efficacy of MLN0128 in the preclinical setting of MCC and delineate downstream targets of mTORC1/2 in MCC cellular systems. MLN0128 significantly attenuates xenograft MCC tumor growth independent of Merkel cell polyomavirus. Moreover, MLN0128 markedly diminishes MCC cell proliferation and induces apoptosis. Further investigations indicate that senescence does not contribute to MLN0128-mediated repression of xenograft MCC tumor growth. Finally, we also observe robust antitumor effects of MLN0128 when administered as a dual therapy with JQ1, a bromodomain protein BRD4 inhibitor. These results suggest dual blockade of PI3K/mTOR pathway and c-Myc axis is effective in the control of MCC tumor growth. Our results demonstrate that MLN0128 is potent as monotherapy or as a member of combination therapy with JQ1 for advanced MCC.

## INTRODUCTION

Merkel cell carcinoma (MCC) is a lethal neuroendocrine cancer of the skin that commonly arises in sun-exposed area, mainly the head and neck regions (1). The incidence of MCC in recent years has been reported to be on the rise globally [1, 2]. Approximately 50% of these patients have metastatic disease at presentation with a 5-year disease-associated mortality rate of 46%, far exceeding that of melanoma [3]. At present there is no effective cure or targeted therapy for these patients [4–6]. Nevertheless, our understanding of the etiopathogenesis of MCC has greatly improved with the detection of Merkel cell polyomavirus (MCV) in 2008 [7, 8]. Although integration of MCV to the host genome has been implicated in the pathogenesis, the exact role of MCV in MCC carcinogenesis still remains an enigma [4–6, 9, 10]. The ‘non-infectious’ etiology of MCC is now being widely accepted as an additional mechanism with frequent reporting of cases on MCV-negative MCC [11–13]. Yet, many of the shared and convergent cell signaling pathways of MCV-positive and MCV-negative MCC are to be delineated [9, 11–14].

The mammalian target of rapamycin (mTOR) is an atypical integrative serine/threonine kinase and a master regulator that governs cell growth, metabolism, proliferation, autophagy, immune function, and apoptosis [15–18]. mTOR kinase resides in two multiprotein complexes, mTOR complex1 (mTORC1) and complex2 (mTORC2), which exhibit different subunit compositions and execute distinct cellular function. Both mTORC1/2 are critical mediators of the phosphoinositide 3-kinase/AKT pathway and thus impact both downstream and upstream of AKT activation. In particular, mTORC1 is responsible for regulating protein synthesis and cell growth, whereas mTORC2 has been shown to phosphorylate and activate AKT. Together, mTORC1/2 control cell growth, metabolism and cell survival [15]. Mounting evidence indicates that PI3K/Akt pathway is inappropriately activated in many human cancers, thus suggesting this as an attractive drug target [16, 19, 20]. Others and we have demonstrated activation of the mTOR-signaling pathway in MCC [21–23]. Interaction between eukaryotic translation initiation factor 4E-binding protein (4E-BP1) and MCV small T antigen is thought to up-regulate mTOR pathway in MCV-positive tumor [21]. Knock-down of mTORC1/C2 activities significantly inhibits cancer cell proliferation in multiple myeloma and other cancers [19, 24] [25–30].

The current focus on mTOR circuitry in cancers came from clinical failures of first generation mTOR inhibitors (rapamycin and its analogs), which mostly inhibit mTORC1 complex, but are ineffective due to feedback activation of mTORC2 complex [15, 16, 31, 32]. The second-generation inhibitors such as MLN0128 target both mTORC1 and mTORC2 and potently inhibit feedback activation of Akt [33]. There is also an emerging consensus that incomplete mTORC1 inhibition (especially in the context of 4EBP1 phosphorylation) is equally or more to blame for the weaker efficacy of rapalogs versus active-site inhibitors. Future studies should help clarify this anomaly. There is an increased interest in the efficacy of MLN0128 since this dual inhibitor has been shown to be potent against several human cancers, including prostate cancer [34] and renal cell carcinoma [35]. Currently MLN0128 is in clinical trial for advanced solid tumors and hematological malignancies (http://ClinicalTrials.gov Identifier NCT01058707). To the best of our knowledge, MLN0128 has not been tested in preclinical setting of MCC.

Since therapy resistance and relapse remains a serious clinical problem, combination therapy is considered a best strategy to combat some of the barriers and escape mechanisms. A growing body of evidence suggests that aberrations in epigenetics play an important role in tumorigenesis [36]. Bromodomain (BRD) and extra-C terminal (BET) domain protein family consists of BRD2, BRD3, BRD4, and BRDT which function as epigenetic readers and gene expression regulators [37, 38]. We have shown a close association between BRD4 and c-Myc expression in MCC [39]. Moreover, we have also demonstrated that JQ1, an inhibitor of BRD4, exerts a potent antitumor activity in MCC in a c-Myc-dependent pathway [40]. Additional evidence in recent years links epigenetic changes in BRD4 as a factor in leukemia, lymphoma, lung adenocarcinoma and melanoma [38, 41–43]. Convergence of c-Myc and PI3K/mTOR pathway is also becoming evident in hematopoietic malignancies [44] and breast cancer [45]. Collectively, these developments suggest that many of the cell signaling pathways in cancer cells are highly interconnected and can converge at mTOR signaling and c-Myc expression. In this study, we show that MLN0128 is a potent inhibitor of mTORC1/2 complex that effectively halts tumor growth, reduce cell proliferation and increase apoptotic cell death. When used along with JQ1 as a combination therapy, it exerted potent anti-tumor effects in MCC.

## RESULTS

### Potent inhibition of tumor growth by mTORC1/2 inhibitor MLN0128

**Figure 1:**
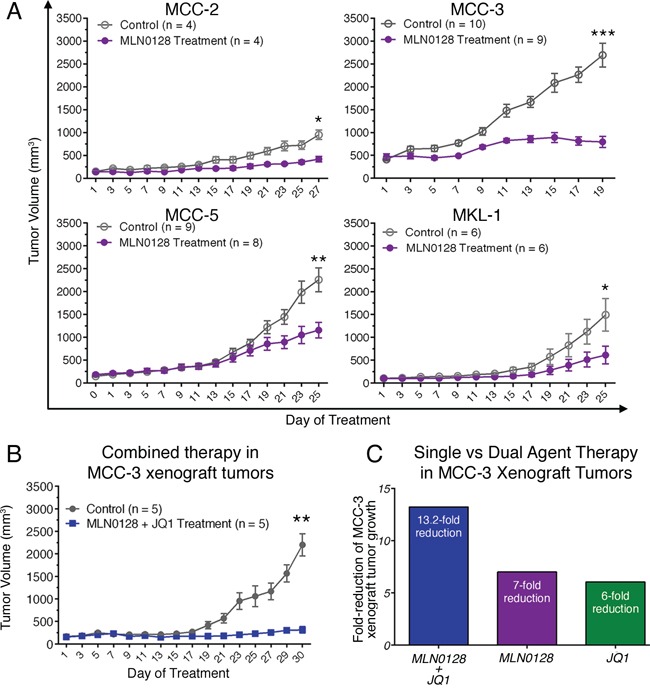
Effect of MLN0128 on MCC xenograft growth **A.** Suppressed xenograft tumor growth in mice treated with MLN0128. Tumor bearing mice were treated with MLN0128 at 1 mg/kg/day or vehicle by oral gavage for seven days followed by a two-day rest. **B.** Repressed MCC-3 xenograft tumor growth upon combined treatment with MLN0128 and JQ1. Tumor bearing mice were treated with MLN0128 or vehicle at 1 mg/kg/day by oral gavage and JQ1 or vehicle at 50 mg/kg/day by i.p. injection for a period of 30 days. **C.** A more effective reduction of MCC-3 xenograft tumor growth in the group treated with combined therapy. Fold-reduction of tumor growth was calculated as average tumor growth of control group divided by average tumor growth of treatment group. Tumor growth was calculated as final average tumor volume minus initial average tumor volume in each group.

The immunodeficient NOD^scid^ gamma (NSG) mouse strain used in this study lacks mature murine T or B cells, and has absent complement activity and NK cell deficiency [21, 22, 46]. We have chosen 3 MCV-negative cell lines developed from the lymph node metastases of individual MCC patients. These MCV-negative cell lines share the characteristic MCC morphology and markers as those in MCV-positive MCC cells [21, 22, 40]. MCV positivity in both our tissues and cell lines was determined by genomic PCR amplification followed by direct DNA sequencing [21, 22]. In this report, we used three of our MCV-negative cell lines (MCC-2, MCC-3 and MCC-5), which were authenticated via STR DNA profiling comparing cell lines with origin tumor tissues (Genetica DNA Laboratories, Cincinnati, OH). MKL-1, a well characterized MCV-positive cell line, was a gift from Dr. Becker (University Clinic Essen, Germany). The tumor engraftment efficiency in MCC was 100% for MCC-2, MCC-3, MCC-5 and MKL-1 cell lines. Characterization of MCC xenograft tumors revealed identical histologic and molecular markers as to the originating cell lines. Treatment with MLN0128 significantly impaired xenograft tumor growth in three different MCC cell lines, albeit in varying degree (Figure 1A). Differential response to MCC-2, MCC-3, and MCC-5 suggest differences in cellular molecular characteristics between cell lines and their inherent susceptibility to mTOR inhibitory therapy. The most significant reduction in tumor volume was observed in MCC-3 xenograft tumors.

### JQ1 augments the anti-tumor effects of MLN0128

**Figure 2:**
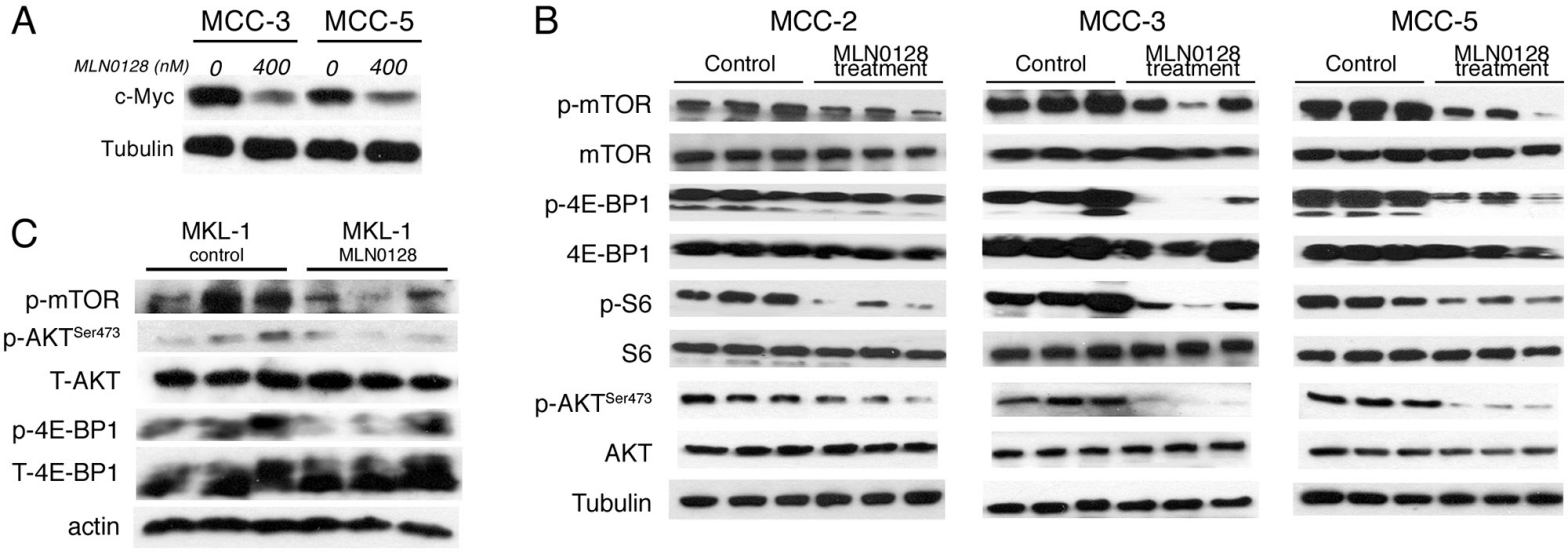
Effect of MLN0128 on c-Myc expression and Akt/mTOR pathway in MCC xenograft tumors **A.** Suppressed c-Myc expression by Western blotting in MCC-3 and MCC-5 cells after MLN0128 treatment. **B.** MLN0128 suppresses phosphorylation of Akt, mTOR and its downstream molecules 4E-BP1 and S6 in MCC-2, MCC-3, and MCC-5 xenograft tumors. Western blot analysis was performed with indicated antibodies. Tubulin was used as a loading control. **C.** MLN0128 suppresses phosphorylation of Akt, mTOR and its downstream molecules 4E-BP1 and S6 in MKL-1 xenograft tumors. Western blotting was performed with indicated antibodies. Actin was used as loading control.

Our earlier studies demonstrated the antitumor effects of JQ1 in c-Myc overexpressing MCC cells [40]. Because MCC-3 and MCC-5 cells overexpress c-Myc, we were intrigued to examine if MLN0128 would also affect c-Myc expression by an interconnected signaling pathway. Accordingly, MCC-3 and MCC-5 cells were treated with MLN0128 at 400 nM for 24 hours. As shown in Figure 2A, c-Myc overexpression was suppressed by MLN0128 in MCC-3 and MCC-5 cells. With this finding, we uncovered an additional transcriptional program that suppresses c-Myc expression [41, 43]. Because c-Myc and mTOR signaling converges at 4E-BP-1 in a c-Myc driven lymphoma mouse model [47], we hypothesized that a combination therapy with JQ1 and MLN0128 might augment anti-tumor effect in our disease model. To this end, we chose MCC-3 xenograft to test our hypothesis since this particular MCC cells have the highest level of c-Myc overexpression. As shown in Figure 1B, a 13.2-fold reduction of tumor growth was observed in MCC-3 xenograft tumors treated with combination therapy. When treated with monotherapies, the anti-tumor effect observed was significantly lower than combination therapy (13.2-fold reduction in combination, vs. 7-fold reduction in MLN0128 alone, vs. 6-fold reduction in JQ1 alone; Figure 1C). Thus, our finding indicates that concomitant inhibition of PI3K/mTOR and c-Myc might be a potential therapeutic strategy in advanced MCC. Additionally, this finding reveals important mechanistic insights into tumor therapeutic resistance development and provides a platform for testing other targeted therapies on PI3K/Akt/mTOR and c-Myc axis to achieve a sustainable therapeutic outcome.

**Figure 3:**
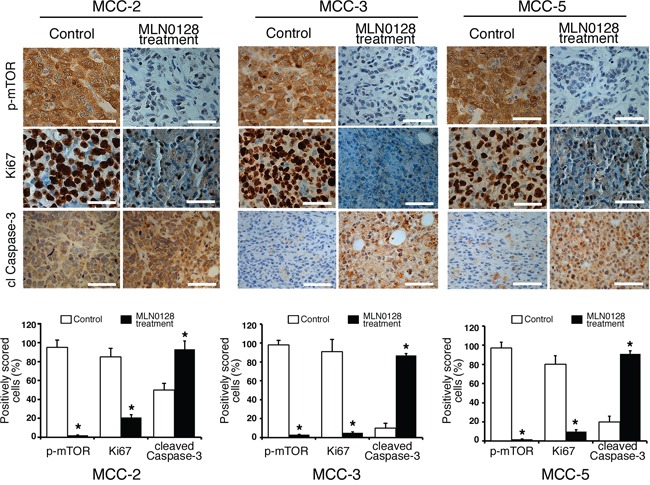
Suppressed cell proliferation and increased cell death in xenograft MCC tumors Immunohistochemical analysis of p-mTOR activity, Ki67+ and cl caspase-3 was performed in fixed xenograft tumors. A significant reduction of Ki67+ positive cells (proliferation) and an increased cl caspase-3 positive cells (cell death) were observed upon MLN0128 treatment. Scale bars, 20 μm (inset). Quantitative cell image analysis was carried out as described in methods section on cells at 400x magnification. Percent positivity (brown reactivity) was calculated from total number of cells in each staining, and control tumors were compared with MLN0128-treated tumors. *p < 0.05 compared with untreated controls.

In our earlier report, we observed that mTOR and downstream targets were activated in MCC tumors [21, 22]. Thus, we carried out subsequent Western blot and immunohistochemical analyses of xenograft to elucidate treatment-related cellular and molecular changes after MLN-128 treatment. As expected, MLN0128 suppressed mTORC1/2 and its downstream effector targets, as evidenced by diminished phosphorylation of mTOR, Akt, 4E-BP1 and S6 kinase (Figure 2B). MCV-positive cell line MKL1 also had similar phospho-protein profile as shown in Figure 2C. Reduced tumor volume in treated group suggested that cell proliferation or cell death might be altered in the xenograft. As shown in Figure 3, Ki67-positive proliferating cells were significantly decreased by MLN0128 at the treatment endpoint. In a similar fashion, increased cell death as demonstrated by cleaved caspase-3 staining was found in the treated group, as shown in Figure 3. As anticipated, p-mTOR was significantly diminished in MLN0128-treated group.

To test if antitumor effects can be observed in MCV-positive MCC, we generated xenograft model using the classic MCC cell line, MKL-1, which harbors MCV. Similar to MKL-1 and other classic MCV-positive MCC cell lines, the MCV-negative cell lines used in this study also grow in cell clusters [40]. As shown in Figure 1A, both MCV-negative and MCV-positive tumors responded to MLN0128 treatment suggesting mTOR is dysregulated in both infectious and non-infectious tumors. Taken together, our results provide strong preclinical evidence implicating mTOR and its downstream targets as important candidate for therapeutic targeting in MCC. This is a meaningful approach since PI3K/Akt/mTOR governs many critical cellular events including metabolism, cell growth, cell cycle, and inflammation. MLN0128, a potent ATP active site inhibitor, is in clinical trials favored over several other dual inhibitors due to its improved pharmacokinetics and long-term metabolic stability [48, 49]. Previous studies have shown mTOR activation via sustained-4E-BP1 phosphorylation by small T antigen of MCV and antitumor effect of mTOR inhibition in MKL-1 cells [21]. In this study, we focused on three MCV-negative MCC cell lines to develop a molecular paradigm identifying major pathways activated and potential therapeutic targets.

### MLN0128 impaired mTORC1 and mTORC2 signaling in MCC cells

**Figure 4:**
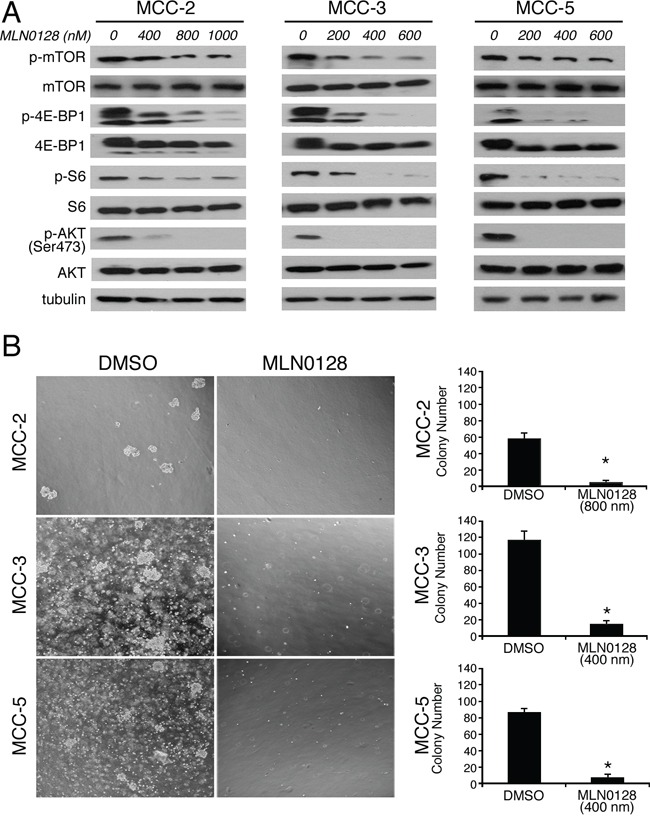
MLN0128 inhibits mTOR pathway activity and colony formation in MCC cells **A.** Suppressed PI3K/mTOR pathway activity upon MLN0128 treatment in MCC cells. MCC cells were treated with MLN0128 for 24 hours at the indicated concentrations and western blotting was performed with indicated antibodies. Tubulin was used as a loading control. **B.** Decreased colony formation in MCC cells treated with MLN0128. Vehicle and MLN0128-treated cells were plated in methylcellulose medium and colonies were counted on Day 21. Left panels show representative images at 40x magnification from different microscopic fields of three MCC cell lines. Right bar graphs indicate the number of colonies at each plating density. Data are presented as the mean ± SEM of triplicate experiments. *p < 0.05 compared with vehicle treated cells.

The development of MLN0128 has facilitated therapeutic targeting of this clinically relevant pathway and downstream components [34]. Furthermore, MLN0128 has been demonstrated to have therapeutic efficacy in several xenograft animal models of human cancers alone or in combination with receptor tyrosine kinase (RTK) inhibitors or PI3K/Akt inhibitor [25–30]. Previously we have shown that the mTOR pathway is up-regulated in MCC tissues and primary MCC cell lines [22]. To further elucidate the activation/inhibition of the mTORC1/2 pathway, we performed *in vitro* culture experiments with MCC cells followed by Western blot analysis. We first treated MCC cells with or without different concentrations of MLN0128 for 24 hours and then examined the total and phosphorylated protein profile of the targeted pathways by Western blotting. Consistent with published reports on other solid tumors, MLN0128 markedly inhibited phosphorylation of both mTOR and its downstream effectors, including 4E-BP1 (Thr37/46) and S6 kinase (Ser235/236) in all three MCV negative MCC cell lines (Figure 4A) [21]. As expected, MLN0128 also abrogated p-Akt activity (Figure 4A) in these cell lines. These results also correlate well with Western blot data shown in Figure 2B and 2C using xenograft tissues.

### Blockade of mTOR pathway inhibited the proliferative capacity of tumor cells

In Figure 1, we attributed phenotypic reduction of tumor volume after mTOR blockade by MLN0128 to decreased cell proliferation and increased cell death within the tumor. To examine these possibilities, we studied *in vitro* effects of mTORC1/2 inhibition by MLN0128 on cell viability and cell proliferation. For this, MCC-2, MCC-3 and MCC-5 cells were treated with increasing concentrations of MLN0128 for 12, 24, 48, and 72 hours, respectively, and cell proliferation were analyzed utilizing CCK-8 assay.

Results from these experiments with three MCC cell lines showed a decreased cell proliferation over a 72-hr period. The half maximal growth inhibitory concentration (GI50) dose was determined by CCK-8 assay in all three MCC cell lines. The GI50 for MCC-2, MCC-3 and MCC-5 cells is 1200 nM, 400 nM and 500 nM, respectively (Data not shown). The underlying mechanism for this variation is not clear. In keeping with this non-responder phenotype, subsequent experiments were carried out at 800 nM for MCC-2 cell line alone. To complement the results from short-term treatments, we performed long-term colony formation assay to determine if the inhibitory effects of MLN0128 were sustained over time. Similarly, MLN0128 significantly decreased the number of MCC cell colonies as compared to that of DMSO controls (Figure 4B). Collectively, our *in vitro* experiments clearly show that blockade of mTOR by MLN0128 inhibits MCC cell growth which partly accounts for the phenotype reduction of tumor. Additionally, the differential response observed among MCC-2, MCC-3 and MCC-5 cells is further suggestive of the heterogeneous nature of this tumor. While all three cell lines share certain common cellular and molecular characteristics, cancer initiation and other changes may be individualistic.

### Cell cycle arrest and augmentation of cell death with mTOR blockade in MCC cells

**Figure 5:**
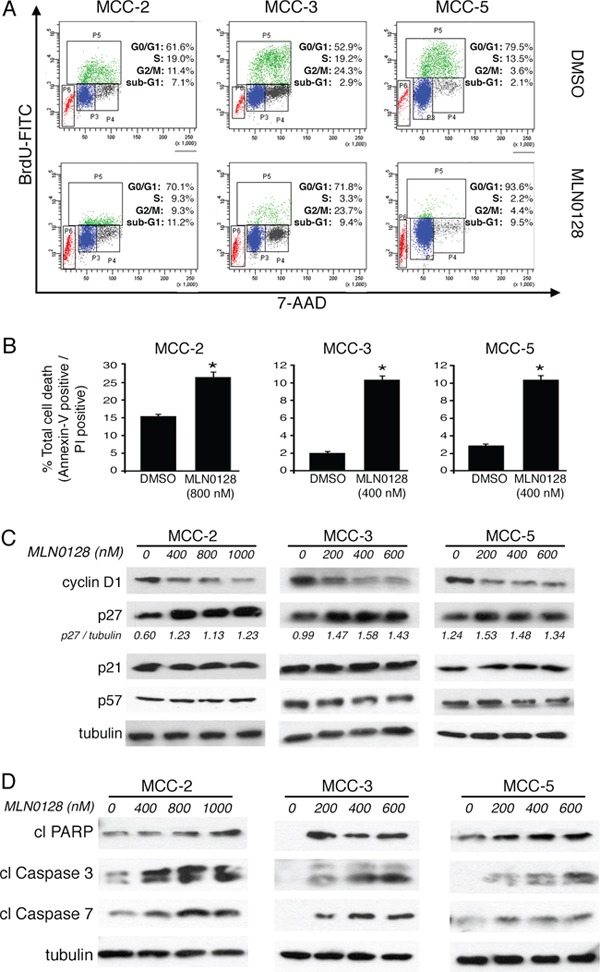
MLN0128 induces G0/G1 cell cycle arrest and cell death in MCC cells **A.** Increased cell cycle arrest in G0/G1 upon MLN0128 treatment. MCC cells were treated with MLN0128 for 24 hours at indicated concentrations, followed by BrdU and 7-AAD staining and flow cytometry analysis. **B.** Increased MCC cell death upon MLN0128 treatment. MCC cells were treated with MLN0128 for 24 hours at indicated concentrations, followed by Annexin V and propidium iodide (PI) staining and flow cytometry analysis cytometry analysis. **C.** Decreased expression of cyclin D1 and increased expression of p27 by Western blotting. MCC cells were treated with MLN0128 for 24 hours at the indicated concentrations. Tubulin was used as a loading control. **D.** Increased cleavage and activation of caspase-3, caspase-7 and PARP by Western blotting. MCC cells were treated with MLN0128 for 24 hours at indicated concentrations. Tubulin was used as a loading control. Data are presented as the mean ± SEM of triplicate experiments. *p < 0.05 compared with control cells.

Because cell proliferation is strictly controlled by cell cycle checkpoints, we analyzed the mTORC1/2 blockade on cell cycle progression using BrdU incorporation labeling method. Cell cycle analysis by flow cytometry showed a significant reduction of cells in the S-phase with a concomitant cell arrest at G0/G1 after MLN0128 treatment in MCC cells (Figure 5A). No such cell cycle arrest events were observed in vehicle alone treated control cells. Additionally, there was a three-fold increase in sub-G1 population in MCC-3 (2.9% to 9.4%) and MCC-5 (2.1% to 9.5%), which are due to apoptotic cell death (Figure 5A). As compared to untreated controls there was only a marginal increased in MCC-2 cells which was less responsive to MLN0128 treatment (7.1% vs 11.2% in treated group) suggesting MCC-2 has a resistant phenotype. When examined by Annexin-V assay for apoptosis, the total apoptotic cell death also found increased significantly in all three cell lines and in particular MCC-3 and MCC-5 (Figure 5B). To identify regulators of cell cycle checkpoint controls perturbed by MLN0128, we examined the expression of cyclin D1, p21, p27 and p57 by Western blot analysis. As shown in Figure 5C, the level of cyclin D1, which regulates the G1/S transition through the cell cycle, was decreased significantly after 24 hr treatment with MLN0128 in all the three MCC cell lines examined. Additionally, MLN0128 treatment resulted in the expression of cell cycle inhibitor p27 without any change in the protein expression of p21 and p57. Taken together, these results suggest that mTORC1/2 inhibitor-associated cell cycle effects were mediated via cyclin D1 down-regulation with concomitant up-regulation of p27. In addition to anti-proliferative effects, TORC1/2 inhibition by MLN0128 induces cell death in other types of cancer.

Furthermore, apoptotic cell death was also markedly increased with the incubation of MLN0128 in the culture. MLN0128 at the concentration of 800 nM increased the fraction of late apoptotic MCC-2 cells from 15.8% to 27.4%. In comparison, MCC-3 and MCC-5 cells were more sensitive even in the presence of 400 nM MLN0128. MCC-3 and MCC-5 cells undergoing cell death increased from 1.9% to 10.8% and from 2.6% to 10.9%, respectively (Figure 5B). Moreover, increased cleavage and activation of PARP, caspase-3 and caspase-7 by Western blotting is indicative of increased apoptotic cell death (Figure 5D).

### Bim up-regulation in apoptotic cells: Role of mTOR blockade

**Figure 6:**
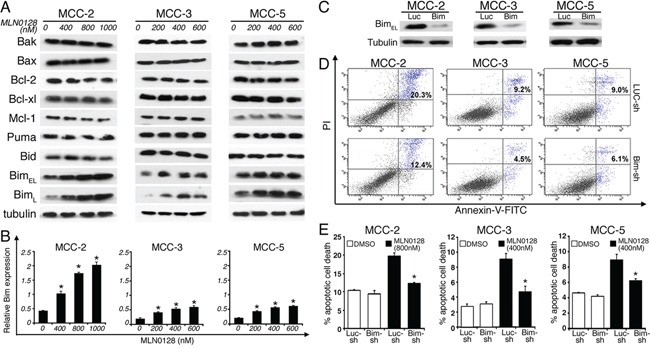
Pro-apoptotic BH3-only protein BIM is up-regulated by MLN0128; knockdown of Bim significantly rescues MLN0128-mediated MCC cell death **A.** Increased Bim expression upon MLN0128 treatment by Western blotting. MCC cells were treated with MLN0128 for 24 hours at indicated concentrations. Tubulin was used as a loading control. **B.** Up-regulated Bim expressions upon MLN0128 treatment by qRT-PCR. MCC cells were treated with MLN0128 for 24 hours at the indicated concentrations. Samples were run in triplicate and normalized to MRPS2 mRNA to determine relative expression (means ± SEM). *p < 0.05 compared with controls. **C.** Decreased Bim expression in MCC cells transduced with Bim shRNA. A non-targeting scramble shRNA served as a control. Tubulin was used as a loading control. **D.** Decreased cell death in Bim-knockdown MCC cells upon MLN0128 treatment. Bim shRNA or non-targeting control shRNA transduced MCC cells were treated with MLN0128 at indicated concentrations, followed by Annexin-V and PI staining and flow cytometry analysis. **E.** A significant reduction in MLN0128-mediated cell death in MCC cells transduced with Bim shRNA compared with non-targeting control shRNA (*p < 0.05). Data are presented as the mean ± SEM of triplicate experiments.

**Figure 7:**
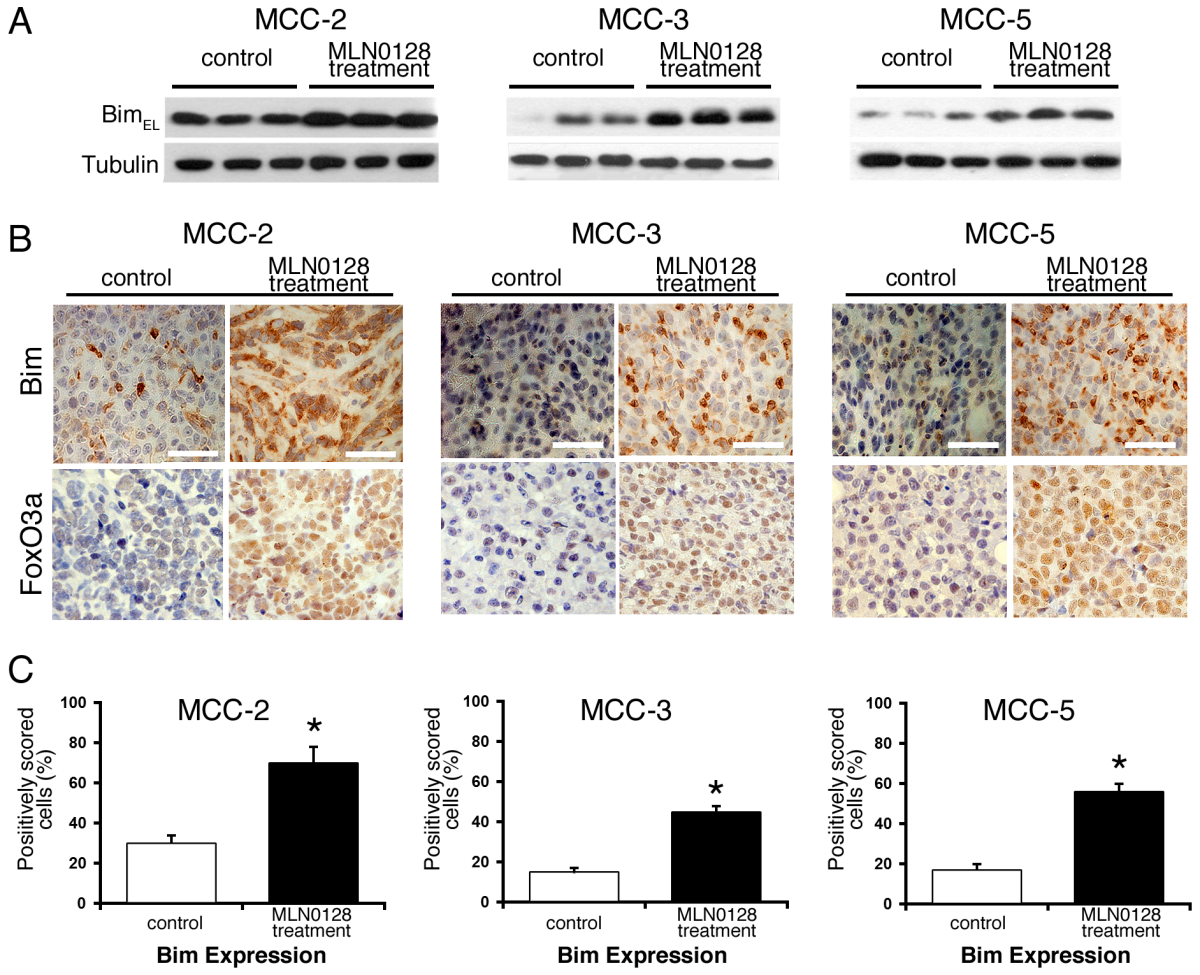
Effect of MLN0128 treatment on Bim and FoxO3a expression in MCC xenografts tumors **A.** Increased expression of Bim in MLN0128 treatment group of xenograft tumor by Western blotting. Tubulin was used as a loading control. **B.** Increased number of Bim positive cells (brown staining) in MLN0128 treatment group. Increased retention of FoxO3a (brown staining) in the nuclei in MLN0128 treatment group. Scale bar in the bottom right panel = 20 μm. **C.** Cells with positive Bim expression were quantified at 400X magnification. The data were presented as the proportion of positively stained cells out of the total number of cells. *p < 0.05 compared with controls.

A large body of literature has shown that Bcl-2 family of proteins contributes significantly to relapse and drug resistance to various cancer therapies [50, 51]. Within the BH3-only proteins of the Bcl-2 family, Bim contributes to resistance to various standard and novel chemotherapeutic agents. Here we demonstrate that treatment with MLN0128 resulted in significant increase in the expression of Bim (Figure 6A and 6B). Western blot analysis of other apoptotic proteins did not show any significant changes among anti-apoptotic Bcl-2 family members (Bcl-2, Bcl-xL, Mcl-1) or pro-apoptotic proteins (Bak, Bax). Similarly, expression of other pro-apoptotic BH3-only proteins (Bid and Puma) also did not alter significantly in all three MCC cells. Similar to MCC, overexpression of Bim in myeloma cells has been associated with poor prognosis [51]. Furthermore, mechanistic studies using BH3 mimetics (ABT-737) also suggested that Bim released from Bcl-2/Bcl-xL accounts for pro-apoptotic activity of Bim. Additionally, Bim inhibited autophagy by sequestering Beclin-1 at microtubules [52]. Together, these results suggest that a Bim-targeting strategy promotes increased apoptotic cell death among cancer cells. These results were further confirmed using gene expression studies. As shown in Figure 6B, mTORC1/2 blockade by MLN0128 resulted in significant increase of Bim mRNA levels in MCC cell lines. Considering that Bim is traditionally characterized as a direct ‘activator’ of Bax/Bak, we silenced Bim expression in MCC cells by Bim shRNA followed by MLN0128 treatment (Figure 6C). As expected, Bim downregulation protected cells from MLN0128 induced apoptotic cell death as examined by Annexin V binding assay (Figure 6D and 6E), confirming the importance of Bim in MLN0128 mediated apoptosis. We extended these studies *in vivo* and results showed increased Bim expression as evidenced from immunohistochemical and Western blotting studies (Figure 7). Together, these results suggest that MLN0128 induced apoptosis is mediated through the up-regulation of BH3-only protein Bim. FoxO class of forkhead proteins are downstream targets of PI3K/Akt pathway [53]. Upon phosphorylation by Akt, FoxO3a is inactivated and accentuated in cytoplasm. Upon PI3K/Akt pathway inhibition (decreased p-Akt), FoxO3a translocates into the nuclei to regulate several cellular events including cell-cycle arrest, cell differentiation, autophagy and apoptosis [54]. To investigate whether similar mechanism is operative in MCC, we examined FoxO3a expression on MCC-2, MCC-3 and MCC-5 xenograft tumors by immunohistochemistry. In agreement, increased FoxO3a staining is detected in the nucleus of MLN0128-treated xenograft tumors, as compared to that in the control group (Figure 7). Interestingly, there are concomitant increased Bim positive cells in the MLN0128 treatment group, indicating a possible mechanism of up-regulation of Bim expression via FoxO3a activation. Further *in vitro* studies are needed to dissect detailed mechanisms in apoptosis triggered by MLN0128 in MCC cells.

### Absence of senescence in MCC xenograft tumors treated with MLN0128

We wondered whether induction of senescence by MLN0128 therapy is a mechanism for repressed xenografts tumor growth. β-galactosidase activity at pH 6.0 has been used as a surrogate marker for senescence, and we examined this aspect in MCC-3 and MCC-5 xenograft tissue sections in the treatment group. First, we experimentally induced senescence in MCF-7 breast cancer cells by etoposide followed by β-galactosidase staining. A distinct blue color staining is indicative of β-galactosidase activity. In comparison, no β-galactosidase positive cells (blue) were detected in both xenograft tumors examined, suggesting senescence is not a mechanism by which MLN0128 inhibits MCC tumor growth (supplementary Figure S1).

## DISCUSSION

Merkel cell carcinoma (MCC) is a lethal neuroendocrine cancer of the skin for which there is no effective cure or targeted therapy [4, 44]. Previously, we demonstrated increased PI3K/Akt/mTOR signaling output in MCC tumors [21, 22]. In the current study, we targeted both PI3K/Akt/mTOR and c-Myc-dependent pathways using *in vivo* and *in vitro* models of MCC. We focused on the therapeutic efficacy of MLN0128, a second generation dual inhibitor of mTORC1/2 and its molecular downstream targets in MCC. Our earlier work uncovered c-Myc overexpression in MCC and demonstrated that JQ1 exerts anti-tumor effect in MCC by suppression of c-Myc [40]. These findings gave us the scientific rationale to focus on mono- and combination therapies, and identify signaling pathways perturbed by MLN0128 and JQ1. We used a combination of cellular, molecular and immunohistochemical techniques to elucidate these molecular drug targets in MCC and the underlying mechanisms involved.

The PI3K/Akt/mTOR signaling axis is central to the carcinogenesis in many human cancers including melanoma, AML, prostate, colon, breast and non-small lung cancer. Hyperactivation of Akt, a serine/threonine kinase, is one of the most commonly activated oncogenic proteins in human cancers and mTORC2 directly phosphorylates Akt at S473 [16, 55]. While examining the role of mTOR in cancer, rapamycin-insensitive mTORC2 complex was discovered and is now being targeted by second generation dual inhibitors such as MLN0128 that directly phosphorylates Akt at S473. We, and others have demonstrated aberrations of mTOR signaling in MCC [21, 22, 56–58]. Thus, targeting PI3K-Akt-mTOR signaling axis through ATP-competitive ‘active-site’ is a promising approach [15, 16, 59–61]. In agreement, MLN0128 blocks phosphorylation of Akt at S473 in a dose dependent fashion, which is further confirmed by *in vitro* experiments such as cell proliferation, colony formation, and most importantly MLN0128 repressed tumor growth in xenograft MCC mouse models. Mimicking human cancers, the MCC cell lines investigated in this study also exhibit heterogeneity and differential responsiveness to MLN0128 both *in vitro* and *in vivo* experiments. Consistent with previous findings, MLN0128 not only down-regulated mTORC1 activity as evidenced from decreased phosphorylation of mTOR, S6 kinase, and 4E-BP1, but also decreased mTORC2 activity via decreased phosphorylation of Akt at Ser473. Though MLN0128 suppressed 4E-BP1 activities in all 3 MCC cell lines investigated, greater inhibitory effect on mTOR was observed with MCC-3 cell line.

To correlate and to better understand the anti-tumor effects of MLN0128 at the cellular level, we studied proteins associated with apoptosis and cell cycle progression. mTOR kinase inhibitors are known to induce cell cycle arrest and cause cytotoxicity, depending on the tumor cell type [62]. Our results have demonstrated a decreased expression of cyclin D1, a known stimulator of G1 cell cycle progression, and overexpression of p27, a cell cycle breaker. No appreciable change was observed in the expression of p21 or p57 after MLN0128 treatment. Thus, MLN0128 selectively disrupts CDK complexes that require p27 as an assembly factor in MCC cells but not those that require p21. Additionally, we have demonstrated increased apoptosis by MLN0128 as evidenced from activation of caspase-3, caspase-7 and PARP in MLN0128-treated group.

Cancer cells evade apoptosis by multiple mechanisms. BH3-only protein Bim and Puma are necessary for MLN0128-induced lymphoid cell death *in vitro* (human cells) and *in vivo* (mice) [19, 25, 30]. Moreover, knockdown of Bim or Puma by RNA interference could completely eliminate MLN0128-induced cell death [30]. In agreement with the pivotal role played by Bcl-2 family proteins in the intrinsic apoptotic pathway, we have found that only Bim is up-regulated at the mRNA and protein level after MLN0128 treatment. Further silencing Bim by shRNA has dramatically protected MCC cells from MLN0128-induced cell death, which implies Bim is a critical factor in MLN0128 mediated cell death. Collectively, these findings highlight the fundamental importance of mTOR in MCC cell proliferation and reinforce the notion that subsets of MCCs are dependent on deregulated mTOR activity. Another corollary of these findings is that therapeutic agents that up-regulated Bim may prime therapy-resistant cells toward cell death. Since a number of pro-apoptotic genes including FasL, Bim and PUMA are regulated by FoxO proteins [63], we examined whether this is the case upon MLN0128 treatment. Our *in vivo* data suggests that FoxO activation as demonstrated by increased nuclear retention of FoxO3a detected in MLN0128-treated xenograft, may be linked to increased Bim expression and increased apoptosis. Further *in vitro* studies are needed to dissect the mechanisms of increased apoptosis upon MLN0128 treatment in MCC. Interestingly, MLN0128 treatment does not induce senescence in MCC xenograft tumors [64].

Interestingly, Moore's group has shown that sustained 4E-BP1 phosphorylation found in MCV-positive MCC is attributed to small T antigen [21]. Together, MCV-positive and MCV-negative cancers may have shared abnormalities in oncogenic signaling though the underlying mechanisms are not fully understood, and targeting mTOR pathway may be therapeutic in MCC regardless of MCV status [40, 65, 66].

Finally, the complexity of PI3K/Akt/mTOR network provides a rationale for combination therapies. Coincident ERK activation is reported in HER2-positive breast cancer cell lines after MLN0128 treatment and better xenograft tumor regression is accomplished by dual mTOR and HER2 blockade [67]. Elevated c-Myc expression in cancers has been shown to be associated with changes in chromatin structure, ribosome biogenesis, metabolic pathways, and apoptosis, among others [66]. Moreover, aberrant epigenetic modifications including DNA methylation and histone modifications have also been shown to contribute to cancer and inflammation [68]. Furthermore, an additive antitumor effect on breast cancer cells was observed when combining mTOR inhibitors with histone deacetylases (HDACs) [45]. Likewise, a significant anti-tumor effect was also evident when MLN0128 was administered as a dual therapy along with JQ1, a BRD4 inhibitor that also targets c-Myc transcriptional amplification in MCC. In spite of these advances, we are just beginning to appreciate the complexity of the mTOR network and downstream targets. Defining these complex and cell-type-specific interactions in MCC cell lines might open the door to new therapeutic strategies.

There are many limitations in extrapolating the laboratory data from xenograft mouse models to the clinic. In human disease, defective tumor immunity is considered one of the hallmarks of cancer. In most cancer types, immune effector cells actively participate in shaping the tumor growth or its elimination. The NSG mouse strain used in this study lacks a functioning immune system, which is a limitation to correctly interpret the data. Several lines of evidence indicate that mTOR pathway regulates both innate and adaptive immune function. In particular, CD4+, CD8+ T-effector cells, Tregs and myeloid suppressor cells are greatly influenced by PI3K/mTOR pathway [69]. While rapamycin is a well-known immunosuppressant, mTORC2 inhibitor can exert a beneficial anti-tumor immunity by modulating Treg-mediated immunosuppression. Furthermore, therapeutic benefits of many conventional and novel therapies relies, at least, in part on stimulating anti-tumor immunity [70].

## MATERIALS AND METHODS

### Reagents

MLN0128 (INK128) was a gift from Dr. Shokat (University of California, San Francisco). JQ1 was purchased from Selleck Chemicals (ThermoFisher, Pittsburgh, PA). Inhibitors were dissolved in sterile DMSO (final concentration < 0.1%), and stored at −80°C in small aliquots. Primary antibodies to mTOR, p-TOR Ser2448, p-4E-BP1 Thr37/46, 4E-BP1, p-S6 Thr389, S6, p-Akt Ser473, Akt, cleaved caspase-3, cleaved caspase-7, cleaved PARP, Bak, Bax, Bcl-2, Bcl-xl, Puma, Bid, Bim and c-Myc were purchased from Cell Signaling (Danvers, MA). Antibodies to cyclin D1, p21, p27, p57, Mcl-1 and tubulin were purchased from Santa Cruz Biotechnology. Anti-FoxO3a antibody was purchased from Abcam (#ab53287, Cambridge, MA). Rabbit polyclonal antibodies to Ki67 (Ab15580) were obtained from Abcam (Cambridge, MA). Additional reagents used in the study include: TransIT-LT1 transfection reagent (Mirus, Madison, WI), puromycin, polybrene, fibronectin and RIPA buffer (Sigma-Aldrich Co., St. Louis, MO). All other biochemical reagents in this study were of analytical grade.

### Establishment of MCC cell lines and tissue culture

MCC cells were maintained in complete RPMI 1640 medium supplemented with 10% fetal bovine serum, 4 mM L-glutamine, 12.5 mM HEPES and antibiotics (Life Technologies, Carlsbad, CA). These cultures were maintained in a dedicated CO2 incubator at 37°C. Cells were fed with fresh medium every other day and cultures were split 1:2 weekly to maintain logarithmic growth. Human embryonic kidney 293T/17 cells (ATCC, Manassas, VA) were cultured in Dulbecco's modified Eagle's medium (DMEM) supplemented with 10% FBS and 5 mg/ml of sodium pyruvate.

### Animal studies: Xenograft transplantation in NSG mice

Immunodeficient NOD/SCID/IL2r-γnull mice (strain #5557) were generated by Jackson Laboratories (Bar Harbor, ME) and their genetic and immunological characteristics published [71]. Five-week old female NSG mice were purchased periodically and maintained in the university animal facility. All animal experiments were performed under a protocol approved by the university's Institutional Animal Care and Use Committee, in accordance with NIH guidelines. MCC cells were prepared from logarithmically growing stock cultures by suspending 2 × 10^7^ cells in 80 μl of media + 120 μl of Matrigel (BD Biosciences, San Jose, CA) and injection sites were prepared by shaving and sterilization with alcohol wipes. Mice received subcutaneous injections of primary human MCC cells on right rear flanks and palpable tumor growth appeared within ∼7 days of inoculation, and treatment protocol began when tumors reached approximately 100 mm^3^ in volume.

### Xenograft drug treatments: *In vivo* study

Tumor-bearing mice were randomly divided into appropriate control and treatment groups (*n* ≥ 4 for each condition) receiving single- or dual-agent therapies. For single-agent therapy, mice received oral administration of 1 mg/kg/day MLN0128 (mTOR inhibitor) or vehicle (5% N-methyl-2-pyrrolidone (NMP), 15% polyvinyl pyrrolidone (PVP) in water). The control and treatment groups received their respective treatments via oral gavage for seven days, followed by two-day rest, for a total of twenty-seven days. For dual-agent therapy, mice received MLN0128 or vehicle by oral gavage and 50 mg/kg/day of JQ1 (10% cyclodextrin in water) or vehicle (10% cyclodextrin in water) by i.p. injection. Drug treatments began when xenograft tumors approached ∼100 mm^3^ in volume. Mice were monitored daily, tumors were measured with digital calipers, and tumor volume was calculated as *L/2* × *W*^2^, where *L* is length and *W* is width as described before [72]. Experimental endpoints were determined by one of the following: (1) completion of treatment course, (2) attainment of tumor burden exceeding 2 cm in any dimension, or (3) further complications affecting animal welfare. Upon reaching experimental endpoints, mice were euthanized humanely, and tumors were excised and dissected for characterization and mechanistic studies. Tumor growth was calculated as average final volume minus average initial volume for each experiment group. The Institutional Committee approved the experimental design and the drug-dosing regimen.

### Immunohistochemistry

Immunohistochemistry was performed on achieved, formalin-fixed, paraffin-embedded tumor tissue. Immunostaining was performed on 5-μm tissue section slides as described previously [21, 22]. Briefly, slides were deparaffinized, dehydrated and processed for antigen retrieval followed by blocking non-specific sites with 10% normal goat serum at RT for one hour. After washes, slides were incubated with primary antibodies for p-mTOR (Ser 2248) (1:100), Ki67 (1:400), cleaved caspase-3 (1:100), FoxO3a (1:50) and Bim (1:100) at 4°C overnight, respectively. After the washing steps, sections were incubated with biotinylated secondary goat anti-rabbit antibody (1:200) for one hour at RT. Subsequently, sections were treated with Vector Elite ABC-HRP reagent (Vector Labs, Burlingame, CA) followed by incubation for 5 min in DAB peroxidase substrate (Vector Labs) for color development. Sections were counterstained with hematoxylin and immunostained slides were viewed on an Olympus BX51 Research System Microscope by 20x and 40x UPlanApo air objective lenses (Olympus America). Images were photographed using a high-resolution interline CCD camera (CoolSNAP, Photometrics) and data acquired with automated microscopy acquisition software (MetaMorph version 7.7; Molecular Devices). Positively stained cells were quantified at × 400 magnification and 5 randomly chosen fields per slide and four slides per group were quantified for each staining. The data were presented as the proportion of positively stained cells over the total number of cells.

### Methylcellulose colony assay

Clonogenic cell formation was assayed by culturing MCC cells in complete methylcellulose (Stem Cell Technologies, Vancouver, BC, Canada) according to the manufacturer's protocol. Briefly, 3000 MCC cells were resuspended in 1 ml complete methylcellulose plated in 35 mm plates and then incubated in a humidified CO_2_ incubator set at 37°C. Colonies (CFU) consisting of at least 40 cells was scored under microscope on day 21 post-seeding.

### Gene expression analysis of bim by qRT-PCR

RNA was isolated from MCC cells with RNeasy kit (Qiagen). cDNA was generated from MCC mRNA using High-Capacity cDNA Reverse Transcription Kit (Life Technologies). RT-PCR was performed in MCC cells as described previously using specific primers for CK7, 18, 19, 20, neuron specific enolase, synaptophysin and Math-1. Quantitative reverse transcription-PCR (qRT-PCR) was performed with a StepOne Plus Real-Time PCR System (Applied Biosystems). The following TaqMan Gene Expression Assays were used: Hs.469658 (Bim) and Hs.211334 (MRPS2). Triplicate runs of each sample were normalized to MRPS2 mRNA to determine relative expression.

### Western blotting

Western blotting was performed essentially as described previously [40]. Briefly, cells were harvested and washed with ice-cold PBS and lysed in 1xRIPA buffer containing 1 mM DTT and Complete Mini EDTA-free protease inhibitor cocktail and incubated on ice for 30 minutes. Cell lysates were clarified by centrifugation at 14,000 rpm for 15 min at 4°C and protein concentration was measured using protein assay kit (Bio-Rad). Lysates (20 μg/lane) were electrophoresed in 8% or 12% SDS-PAGE gel electrophoresis and transferred onto PVDF membrane by a semi-dry system (Bio-Rad) and reacted with optimal concentrations of specific primary antibodies at 4°C overnight followed by secondary antibodies conjugated with HRP (Cell Signaling) for 1 hour at room temperature. Visualization of immunoreactive proteins was achieved with ECL chemiluminescence detection system (Millipore, Billerica, MA). Alpha-tubulin was used as loading control. Similarly, xenograft tumor tissues harvested from mice were homogenized in 2% SDS lysis buffer and processed for Western blotting as described above. Immunoblotting data represent contiguous lanes.

### Cell proliferation and cell viability

Cell proliferation and cell survival was assessed using Cell Counting Kit-8 (Sigma-Aldrich) according to the manufacturer's instructions. In brief, MCC cells were plated at 10,000 cells per well in a 96-well plate, allowed to attach overnight, and then exposed to various concentrations of MLN0128. Plates were incubated for 12, 24, 48 and 72 hours at 37°C in a CO2 incubator. At the end of incubation period, CCK-8 (10% v/v) was added to each well and incubated for another 4 hours at 37°C. The absorbance at 450 nm was measured using a microplate reader.

### Determination of apoptosis by flow cytometry

Apoptotic cell death was determined using Annexin V-FITC apoptosis detection kit (BD Biosciences). Briefly, MCC cells were plated in 6 well plates (1 × 10^6^ per well) and treated with mTOR inhibitors for 24 hours. Cells were harvested, washed in PBS and stained with Annexin-V and propidium iodide (PI) cocktail as per the manufacturer's protocol. Control cells were incubated in 1x binding buffer and stained with Annexin-V or PI alone. Cells were analyzed in FACSAria (BD Biosciences) within an hour of staining. Cell death was scored by the following criteria set by appropriate gating: (a) early apoptotic cells (PI negative, FITC Annexin-V positive), (b) late apoptotic cells or dead cells (double positivity for both FITC Annexin-V and PI), and (c) live cells (double negative for Annexin-V and PI) and statistical analysis was performed using FACSDiva software.

### Cell cycle analysis

For cell cycle analysis, MCC cells were seeded at a cell density of 1 × 10^6^ per well in 6-well plates, treated with mTOR inhibitors for 48 hours, and labeled with 10 μM bromodeoxyuridine (BrdU) for 2 hours as described before [40]. BrdU incorporation in these cells was detected using Alexa Fluor 488-conjugated mouse anti-BrdU antibody followed by 7-AAD staining as per the manufacture's protocol (BD Biosciences). At least 20,000 cells were acquired for each treatment condition and cell cycle analysis was performed in the FACSAria flow cytometer using FACS-Diva software. The cell cycle distribution was reported as the percentage of cells in the G0/G1, S, and G2/M populations.

### Gene silencing of bim by shRNA method

Lentivirus-based short hairpin RNA (shRNA) system is an efficient gene delivery tool to functionally silence genes in mammalian cells. Lentivector containing shRNA specific for Bim (TRCN0000029119) and non-targeting plasmid pLKO.1 scramble shRNA (plasmid #1864; negative control vector) were purchased from Sigma-Aldrich and Addgene (Cambridge, MA) respectively. ShRNA-encoding lentivirus was packaged in 293T/17 cells to generate viral particles as described before [73]. Viral supernatants were collected from these cells 48 hours after transfection. Viral particles were recovered after ultracentrifugation and resuspended in PBS. After spinocculation, MCC cells were transduced with lentivirus supernatant for 48 hours in fibronectin-coated 6-well plates in the presence of 8 μg/ml polybrene. Since lentiviral shRNA particles also encode for a puromycin resistance gene for transduction selection, cells were washed and grown in culture media containing 2 μg/mL puromycine dihydrochloride for an additional 72 hours. The remaining successfully transduced cells were allowed to recover and proliferate for at least 2 days before any experimental procedure.

### Assessment of cell senescence

Senescence was assessed using a commercially available beta-galactosidase staining kit according to the manufacturer's protocol (Cell Signaling Technology, Danvers, MA, # 9860). Briefly, 8-μm frozen tissue sections were made and air dried for 10 minutes. The slides were fixed for 15 minutes in 1x fixative solution (formaldehyde-glutaraldehyde mix), followed by PBS washes, and incubated overnight with beta-galactosidase staining solution in a dry incubator at 37°C with no CO_2_ supply to prevent false positivity. Sections were viewed under microscope for blue color development and were photographed for image analysis. As a positive control, MCF-7 breast cancer cell line (ATCC, Catalog #HTB-22) was experimentally induced for senescence by incubating with 12.5 μM of etoposide for 24 hours. Next, etoposide was removed and cells were cultured in fresh media for 4 days. Changes in morphology were assessed daily and on the 5^th^ day cells were fixed followed by staining per manufacturer's instructions. Subsequently, both MCF-7 cells and tissue sections were counterstained with nuclear red to visualize nuclei (Sigma-Aldrich, #N3020, St Louis, MO). Development of distinct blue color is indicative of senescence.

### Statistical analysis

All measurements were made in triplicate, and all values are represented as mean ± SEM. Statistical analysis was performed with Student's *t*-test, one-way analysis of variance (ANOVA), or Mann-Whitney *U* test. *P* values < 0.05 were considered statistically significant.

## CONCLUSION

To our knowledge, this is the first study demonstrating the therapeutic efficacy of mTORC1/2 blockade on MCC by MLN0128. We also demonstrate dual inhibition of mTOR and BRD4 led to a better anti-tumor effect in c-Myc overexpressing MCC tumors. As PI3K and mTOR pathways as well as their close proximity to immunotherapies are defined, effective therapies can be developed for MCC. Future cancer genomic studies may lead to better cancer biomarkers and evidence-based patient management.

## SUPPLEMENTARY FIGURE


