# Supplementary material for: Dual mTOR inhibitor MLN0128 suppresses Merkel cell carcinoma (MCC) xenograft tumor growth
Source: Oncotarget. 2015 Oct 23;7(6):6576–92. doi: 10.18632/oncotarget.5878 (PMC4872734; doi:10.18632/oncotarget.5878)
Supplement: Supplementary file 1 [file oncotarget-07-6576-s001.pdf]

SUPPLEMENTARY FIGURE

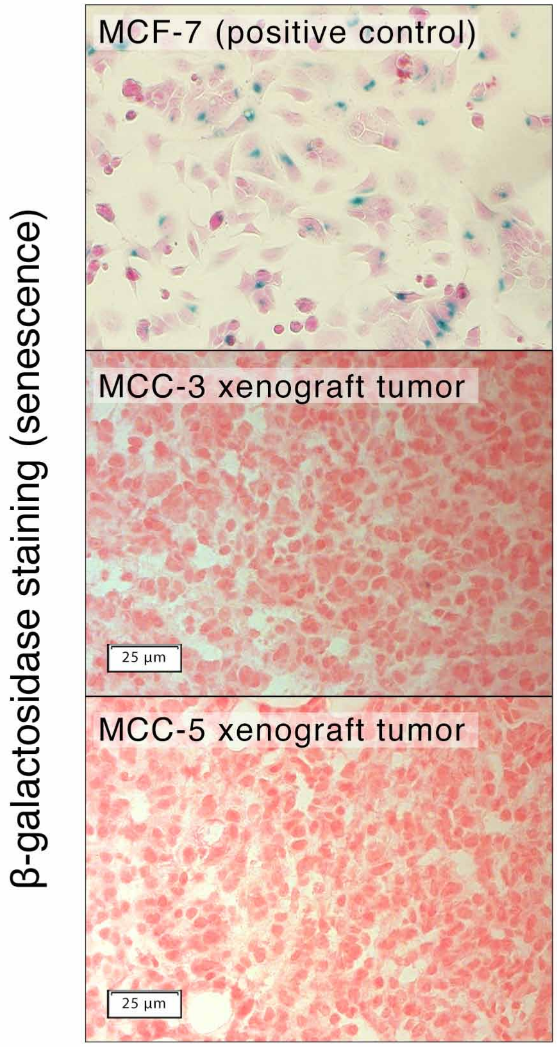

Supplementary Figure S1: Absence of senescence in MCC xenograft tumors treated with MLN0128. Frozen MCC xenograft tissue sections were assessed for beta-galacatosidase activity at pH 6.0 as a marker for senescence. MCF-7 breast cancer cells were experimentally induced as a positive control for senescence. MCF-7 cells were incubated with 12.5 µM etoposide for 24 h, washed and allowed to recover in fresh media for 4 days, and then assessed for beta-galactosidase staining as detailed in the methods section. Both MCF-7 cells and xenograft tissue sections were counterstained with Nuclear Red. Distinct blue color is indicative of beta-galactosidase activity.
